# Predictive model for the risk of paediatric intensive care utilization in children with medical complexity: A longitudinal retrospective cohort study

**DOI:** 10.1111/nicc.13180

**Published:** 2024-10-08

**Authors:** Bibiana Pérez‐Ardanaz, Laura Gutiérrez‐Rodríguez, Alberto José Gómez‐González, José Miguel Morales‐Asencio, Antonio Montero‐García, Álvaro León‐Campos

**Affiliations:** ^1^ Department of Nursing, Faculty of Health Sciences Universidad de Granada Granada Spain; ^2^ Instituto de Investigación Biomédica de Málaga (IBIMA‐Bionand) Málaga Spain; ^3^ Department of Nursing, Faculty of Health Sciences Universidad de Málaga Málaga Spain; ^4^ Centro de Emergencias Sanitarias 061, Servicio Provincial de Málaga Málaga Spain

**Keywords:** children, health resources, multiple chronic conditions, nursing, paediatric critical care

## Abstract

**Background:**

Children with medical complexity (CMC) are at increased risk of admission in intensive care. Despite improvements in mortality rates, there remains a burden of morbidity, long‐term health care needs and hospital readmissions. Beyond clinical factors, socio‐demographic determinants could impact utilization of acute services.

**Aim:**

To identify risk factors that can differentiate CMC who are admitted to the paediatric intensive care unit (PICU).

**Study Design:**

A 6‐year longitudinal retrospective cohort study evaluated clinical, socio‐demographic and health care utilization.

**Results:**

A total of 248 CMC were included, with a median age of 13 years (9.75–17.00). Intensive care admission rate was 47.2%. The risk of PICU admission was higher for children undergoing surgical interventions (HR = 1.58, 95% CI 1.34–1.86, *p* < .001) and those using medical devices (HR = 1.81, 95% CI 1.54–2.13, *p* < .001). Mother's higher educational level was a protective factor (HR = 0.66, 95% CI 0.55–0.79, *p* < .001). Multivariable analysis revealed significant associations between risk of admission and the presence of malignancy, comorbidities, home medical devices, surgical procedures and higher health care utilization. Children's age and higher maternal educational level acted as protective factors.

**Conclusion:**

Socio‐demographic factors should be considered in the provision of care to CMC. Individualized assessments to guide supportive interventions adapted to socio‐economic factors may prevent PICU admissions.

**Relevance to Clinical Practice:**

This study highlights the importance of integrating individualized assessments of socio‐demographic risk factors, such as maternal educational level, into the clinical practice of paediatric nurses. Moreover, targeted interventions, including educational resources and community support programmes, may optimize care.

**What is known about the topic**

- Children with medical complexity are more likely to be admitted to intensive care units.
- Socio‐demographic factors of the child and their families can impact on the outcomes that arise during health care.

**What this paper adds**

- This study provides insights for health care professionals to proactively identify patients at risk of admission to the paediatric intensive care unit (PICU).
- Individualized assessments promote a more holistic approach to care for children with medical complexities.
- The findings suggest interventions to reduce PICU admissions through optimized care and resource utilization.

## 1 INTRODUCTION

The prevalence of children with medical complexity (CMC) among hospitalized patients,^1^ and particularly those admitted to the paediatric intensive care unit (PICU), is on the rise. CMC is defined as ‘any medical condition that can be reasonably expected to last at least 12 months (unless death intervenes) and which involves either several different organ systems or one organ system severely enough to require specialty paediatric care and probably some period of hospitalization in a tertiary care centre.’^2^

## 2 BACKGROUND

These conditions are responsible for a disproportionate use of ICU resources in paediatric hospitals,^3^ often resulting in extended hospital stays, unplanned readmissions and mortality.^4^ CMCs have a three times higher risk of suffering from an acute episode requiring admission to the PICU, and may represent up to more than 70% of admissions to the PICU (almost half of all unplanned admissions).^4^ Furthermore, they have a higher risk of mortality and represent most of the length of stay (LOS) compared with the rest of the paediatric population.^3^, ^4^, ^5^, ^6^ Moreover, additional challenges increase the risk of complications during PICU hospitalization, such as heterogeneity of chronic conditions, extensive medication requirements, altered anatomy or physiology, and developmental needs.^7^

Nevertheless, mortality rates among children admitted to the PICU have enhanced because of improvements in health care organization and a better understanding of chronic diseases.^7^, ^8^ However, survival is at the cost of increased morbidity rates,^7^ considerable long‐term health care^9^ and hospital readmission.^7^

Acquired functional impairment (morbidity) in the PICU survivor population ranges from 4.6% to 36% at discharge and persists at 10%–13% beyond 2 years after discharge.^10^ Certain admission diagnoses, younger age and the need for mechanical ventilation are associated with better survival rates, but they trigger additional morbidity associated with a widespread range of temporary or long‐term impairments.^9^

Nonetheless, there are further factors that go beyond the clinical risk factors, such as socio‐demographic determinants of the child and their families that may influence the final outcomes throughout the care process.^8^ The comprehension of these interactions throughout the follow‐up of CMC could provide a framework to deploy appropriate prevention and reduction measures for admissions, acquired morbidity and mortality. To our knowledge, no previous predictive models aimed at this purpose are available because there are few longitudinal studies in PICUs with CMC that simultaneously evaluate clinical, demographic and health care use factors.

## 3 THE STUDY

### 3.1 Aim

The aim of this study was to identify risk factors able to differentiate CMC who are likely to require admission to the PICU.

## 4 METHODS

### 4.1 Study design and setting

This was a longitudinal study based on the CMC included in a previous cross‐sectional study. ^11^ A six‐year follow‐up retrospective cohort was designed for this purpose. The study followed the Strengthening the Reporting of Observational Studies in Epidemiology (STROBE) reporting guidelines.

This study was conducted at a blinded reference hospital in Spain.

### 4.2 Inclusion or exclusion criteria

**FIGURE 1:**
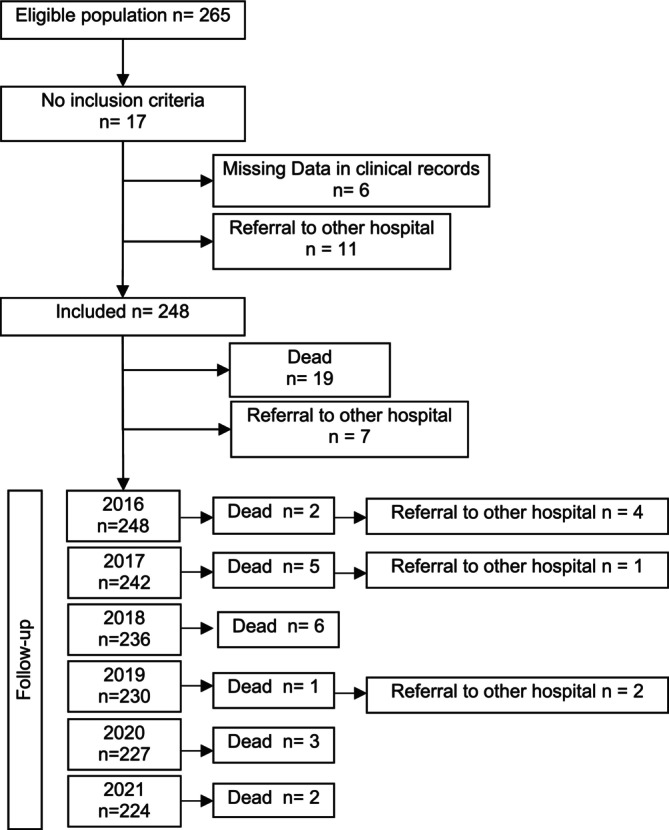
Participant flow chart.

Participants were children and adolescents aged under 18 years who had complex chronic conditions (CCCs) according to the criteria proposed by Feudtner et al.: ‘Child or young adult from one month of age with a medical condition reasonably expected to last at least 12 months (unless death intervenes) and involves several different organ systems or one system severely enough to require specialty paediatric care and some period of hospitalisation in a tertiary care hospital.’^2^ This classification has nine organ system‐based CCC types previously identified by the International Classification of Diseases 10th Revision (ICD‐10).^2^ The 6‐year follow‐up showed that some subjects exceeded the upper age limit of the inclusion criteria. Nevertheless, because many of them continued to receive care from paediatric services or in a transitional process between adult and paediatric services, we decided to include them in the final sample. The flow chart is detailed in Figure 1.

This study is a continuation of an initial study focusing on children who already had CCCs at the start. Therefore, the study did not include new cases of children developing CCCs during the follow‐up period; it tracked the initial cohort of children with CCCs over the 6‐year period.

Sample size calculation was conducted during the design of the previous cross‐sectional study,^11^ from which this longitudinal study follows. Based on preliminary data and statistical considerations, including a significance level (α) of 0.05, a power (1‐β) of 0.80 and an expected moderate to large effect size in PICU admission rates, a sample size of approximately 250 children was determined to be adequate. This calculation was maintained for the longitudinal study to ensure consistency and sufficient statistical power, as it follows the same patient cohort.

### 4.3 Data collection

A 6‐year time frame was used from January 2015 to December 2021 drawn from the electronic records of a tertiary care children's hospital that is a referral centre from the National Health Care Service (Spain). The main outcome variable was PICU admission. Clinical and socio‐demographic variables were recorded. Health care use apart from PICU (admission and length of PICU stay) was verified, such as emergency services frequentation, surgical procedures, hospital admissions, length of hospital stay, use of medical devices at home and ambulatory consultations. Additionally, the use of paediatric palliative care (PPC) services was identified (this unit was established at the hospital in 2018). Mortality was checked using the date of death. The survival time frame was determined from the date of diagnosis until the last follow‐up date. Additionally, to account for the time since exposure to CCC, we calculated the duration from the initial diagnosis of CCC to the outcome events and included this variable in our regression models and survival analyses. This ensures that the time factor is appropriately considered in our statistical assessments.

COVID‐19 infection was recorded to examine its influence as a potential competitive PICU use risk factor.

Due to the low frequency of some categories, CCCs were recoded into three categories: neurological‐congenital‐genetic condition, acquired condition and malignancy^12^; scheduled surgery and urgent surgery into surgical intervention; and the number of comorbidities was classified into three categories (none; 1–2 comorbidities; >2 comorbidities).

### 4.4 Data analysis

Data were summarized as frequencies and percentages for categorical variables and as medians with quartile 1 and 3 for continuous variables. The normality of continuous data was assessed using the Shapiro–Wilk test. As most data did not follow a normal distribution, non‐parametric tests were used. The Wilcoxon rank‐sum test was used to compare non‐normally distributed data, and proportions were compared using the chi‐squared test or Fisher's exact test as appropriate.

Analysis of covariance (ANCOVA) was performed to investigate the relationship between PICU admission and health care service usage while controlling for potential confounding factors. The Kaplan–Meier method was used to determine unadjusted rates of 6‐year survival. Differences in survival between groups were evaluated using the log‐rank test. A Cox proportional hazards model was used to identify risk factors independently associated with PICU admission, calculating hazard ratios (HRs) to quantify the relative risk. HRs were interpreted with confidence intervals to assess their significance and magnitude, and the proportional hazards assumption was verified using survival graphs. Additionally, odds ratios (ORs) were used in logistic regression analyses for specific subgroups, such as surgical interventions and the use of the PPC unit. The analyses included socio‐demographic, clinical and health care utilization (HCU) variables. All analyses were performed using JAMOVI 2.3.38.

### 4.5 Ethical considerations

This study used data from clinical records and did not use direct data collection from patients or their families; therefore, the ethics committee of Malaga considered it exempted from the need for approval and informed consent, confirming the validity of the previous study's approval (No.0655‐N‐16) (date of consultation: 02/12/2021).

## 5 RESULTS

The sample consisted of 248 children, of which 55.8% (*n* = 139) were male. Their median age was 13 years (9.75–17.00). CCC groups were distributed in 59.7% (*n* = 148) for neurological‐congenital‐genetic condition, 24.6% (*n* = 61) for acquired condition and 15.7% (*n* = 39) for children with malignancy. Regarding mortality, 19 children (1.1% of the sample) died during the follow‐up period.

### 5.1 PICU admission rates

The median survival time from diagnosis was 7.05 (7.05–7.05) and slightly higher in children who did not require admission to PICU than in those who were admitted (effect size: 0.08; *p* < .001). These differences were also significant in the non‐adjusted mortality analysis (HR: 6.98 95% CI: 5.89–8.27; *p* < .0001).

Table 1 displays the characteristics of the sample according to admission to the PICU.

**TABLE 1:** Demographic and clinical characteristics of participants, by paediatric intensive care unit admission.

PICU admission	Median (Q1–Q3) or *n* (%), children (*n* = 248)		
Characteristics	NO PICU (*n* = 131)	PICU (*n* = 117)	*p*
Age (years), median (95% CI)^a^ children	14.00 (10–17)	12.44 (9–16)	<.001
Covid, *n* (%)^b^	7 (41.2)	10 (58.8)	.319
Organ transplantation, *n* (%)^b^	4 (36.4)	7 (63.6)	.263
Mortality, *n* (%)^b^	5 (25)	15 (75)	.009
CCC classification, *n* (%)^b^			
Malignancy	9 (23.1)	30 (76.9)	<.001
Acquired condition	35 (57.4)	26 (42.6)	
Congenital‐neurological condition	87 (58.8)	61 (41.2)	
Comorbidities Group, *n* (%)^b^			
None	20 (57.1)	15 (42.9)	.851
1–2 comorbidities	61 (51.7)	57 (48.3)	
>2 comorbidities	50 (52.6)	45 (47.4)	
Comorbidities number, means (SD)^a^	2.18 (1.66)	2.18 (1.95)	.360
Medical devices at home, *n* (%)^b^	24 (35.8)	43 (64.2)	.001
Oxygen therapy	20 (27.0)	54 (71.0)	<.001
Mechanical ventilation	0	31 (100)	<.001
Enteral feeding	13 (22.8)	44 (77.2)	<.001
Urinary catheter	7 (23.3)	23 (76.7)	.001
Gastrostomy	9 (28.1)	23 (71.9)	.003
Tracheostomy	0	11 (100)	<.001

*Note*: *p* level confidence.

Abbreviations: CCCs, complex chronic conditions according to the Feudtner et al. classification; PICU, paediatric intensive care unit.

^a^ Wilcoxon rank‐sum test.

^b^ Chi‐squared test‐Fisher's exact test.

During the follow‐up period, 47.2% of the sample (*n* = 117) were admitted to PICU. The median use of health resources was higher among those who were admitted to the PICU—135.5 (85–176) than those who were not admitted 95 (60–137; effect size: 0.28; *p* < .001). Table 2 shows the use of health resources according to PICU admission during the follow‐up period.

**TABLE 2:** Utilization of health care resources 2015–2021 by paediatric intensive care unit admission.

	2015 (*N* = 248)		2016 (*N* = 248)		2017 (*N* = 242)		2018 (*N* = 236)		2019 (*N* = 229)		2020 (*N* = 227)		2021 (*N* = 224)	
PICU admission	NOT (*N* = 179)	YES (*N* = 51)	NOT (*N* = 186)	YES (*N* = 62)	NOT (*N* = 220)	YES (*N* = 21)	NOT (*N* = 221)	YES (*N* = 15)	NOT (*N* = 213)	YES (*N* = 16)	NOT (*N* = 215)	YES (*N* = 12)	NOT (*N* = 215)	YES (*N* = 9)
Health resources^a^	I‐J (95% CI)	*p*	I‐J (95% CI)	*p*	I‐J (95% CI)	*p*	I‐J (95% CI)	*p*	I‐J (95% CI)	*p*	I‐J (95% CI)	*p*	I‐J (95% CI)	*p*
Hospital A&E visits	−0.4 (−1.2;0.3)	.215	−0.7 (−1.6; −0.2)	.121	−0.6 (−1.5;0.1)	.105	−0.4 (−1.1;0.3)	.280	−0.3 (−1.1; 0.4)	.363	0.32 (−0.4; 0.4)	.881	−0.5 (−1.1;0.5)	.072
Programmed hospital Ad	−9.6 (−1.6; −0.3)	.003	−0.8 (−1.3; −0.3)	.002	−0.2 (−0.5;0.2)	.071	−0.2 (−0.6;0.1)	.146	−0.3 (−0.1;0.1)	.578	−0.2 (−0.6; 0.2)	.277	0.0 (−0.2;0.2)	.822
Urgent hospital Ad	−0.7 (−1.0; −0.4)	<.0001	−1 (−1.3; −0.7)	<.0001	−0.5 (−0.7; −0.2)	.001	−0.3 (−0.5; −0.1)	.001	−0.2 (−0.3; −0.1)	.005	−0.1 (−0.2; 0.5)	.193	−0.2 (−0.3;0.0)	.015
Length of hospital stay	−14.5 (−20.2; −8.8)	<.0001	−15.7 (−20.7; −10.6)	<.0001	−5.2 (−7.6; −2.8)	<.0001	−3.6 (−5.8; −1.4)	.002	−2.1 (−3.7; −0.5)	.009	0 (−1.5; 1.4)	.956	−1.8 (−3.2; −0.4)	.011
Ambulatory consultations	−3.4 (−6.6; −0.2)	.036	−4.2 (−6.8; −1.5)	.003	−3.2 (−5.5; −1.0)	.005	−2.4 (−5.1;0.2)	.071	−1.2 (−4.5;2.0)	.455	−1.1 (−4.2; 1.9)	.462	−2.3 (−5.4;1.0)	.142
Total health care contacts	−8.9 (−14.2; −3.6)	.001	−11.5 (−15.5; −7.5)	<.0001	−6.6 (−10; −3.3)	<.0001	−5.8 (−9.7; −1.9)	.003	−2.2 (−5.9; 1.3)	.221	−1.3 (−4.7;2.1)	.451	−2.9 (−6.6; 0.8)	.129

Surgery contacts^b^	*N* (%)		χ^2^ *p*	*N* (%)		χ^2^ *p*	*N* (%)		χ^2^ *p*	*N* (%)		χ^2^ *p*	*N* (%)		χ^2^ *p*	*N* (%)		χ^2^ *p*	*N* (%)		χ^2^ *p*
PICU admission	NOT	YES		NOT	YES		NOT	YES		NOT	YES		NOT	YES		NOT	YES		NOT	YES	
Planned surgery	12 (29.3%)	29 (70.7%)	13.512 <.0001	19 (36.5%)	33 (63.5%)	7.001 .008	19 (46.3%)	22 (53.7%)	1.081 .299	11 (47.8%)	12 (52.2%)	0.422 .516	9 (52.9%)	8 47.1%	0.047 .828	14 (50.0%)	14 (50.0%)	0.392 .531	7 (53.8%)	6 (46.2%)	0.013 .910
Urgent surgery	3 (21.4%)	11 (78.6%)	6.859 .009	3 (25%)	9 (75%)	3.917 .048	0 (0%)	0 (0%)	0 .221	1 (33.3%)	2 (66.7%)	0.535 .465	1 33.3%	2 (66.7%)	0.602 .438	1 (20.0%)	4 (80%)	2.610 .106	2 (100%)	0 (0%)	1.627 .202
Total surgery	15 (29.4%)	36 (70.6%)	17.540 <.0001	21 (33.9%)	41 (66.1%)	11.914 .001	19 (46.3%)	22 (53.7%)	1.081 .299	12 (48.0%)	13 (52.0%)	0.438 .508	10 (50.0%)	10 (50.0%)	0.549 .459	15 (38.4%)	18 (61.6%)	0.737 .391	9 (57.1%)	6 (42.9%)	0.019 .890

*N* (%)		χ^2^ *p*	*N* (%)		χ^2^ *p*
NOT	YES		NOT	YES	
14 (50.0%)	14 (50.0%)	0.392 .531	7 (53.8%)	6 (46.2%)	0.013 .910
1 (20.0%)	4 (80%)	2.610 .106	2 (100%)	0 (0%)	1.627 .202
15 (38.4%)	18 (61.6%)	0.737 .391	9 (57.1%)	6 (42.9%)	0.019 .890

*Note*: Unit established in 2018.

Abbreviations: A&E, accident and emergency; Ad, admission; ICU, intensive unit care; NA, not available; PICU, paediatric intensive care unit; PPC, paediatric palliative care.

^a^ ANCOVA (Student's *t*‐test).

^b^ Chi‐squared test.

### 5.2 Surgical interventions

Among the children in the study, 52.4% (*n* = 130) underwent scheduled surgery. Of the scheduled surgeries, 56.2% (*n* = 73) resulted in admission to the PICU (χ^2^ = 8.05; *p* = .005; OR: 2.08; 95% CI: 1.25–3.45). Regarding urgent surgery (*n* = 36; 14.5%), 75.0% (*n* = 27) were referred to the PICU (χ^2^ = 12.7; *p* < .001; OR: 3.99; 95% CI: 1.79–8.89) (Table 2).

### 5.3 Paediatric palliative care unit usage

From the total sample, 26 children (10.5%) used the PPC unit, of which 69.2% (*n* = 18) were admitted to the PICU (χ^2^ = 5.46; *p* = .019; OR: 2.74; 95% CI: 1.15–6.58).

### 5.4 Multivariate Cox regression analysis

The multivariate Cox regression model, constructed to determine the risk analysis for PICU admission, showed that the variables ‘CCC classification’,” ‘surgical intervention’, ‘transplant’, ‘any medical device’, ‘mother's educational level’, ‘child age’, ‘comorbidities’ and ‘total health care frequentation’ collectively have a predictive capacity of 21.3% for PICU admission. The adjusted HR values for admission are presented in Table 3.

**TABLE 3:** Hazards regression model.

			HR (95% CI), *p*
CCC classification	Malignancy	237 (14.6)	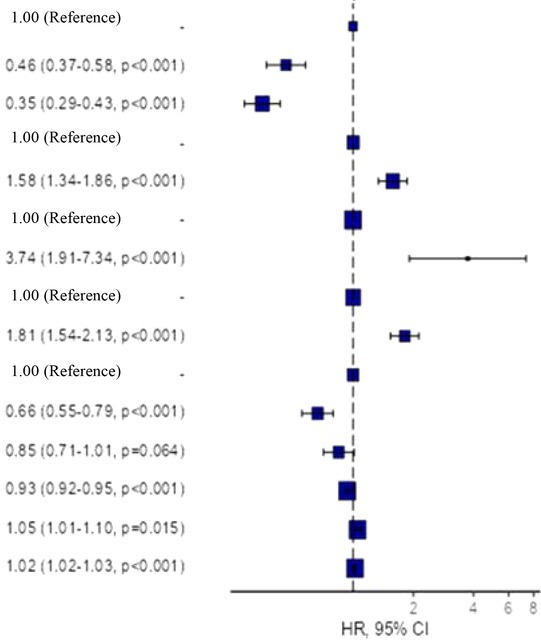
	Acquired condition	406 (25.1)	
	Congenital‐neurological	975 (60.3)	
Organ transplantation	None	1605 (99.2)	
	Yes	13 (0.8)	
Surgical intervention	None	699 (43.2)	
	Yes	1605 (56.8)	
Any medical device	None	1176 (72.7)	
	Yes	442 (27.3)	
Mother educational level	Illiteracy or primary school	573 (35.4)	
	Secondary or high school	557 (34.4)	
	University grade	488 (30.2)	
Child age	Mean (SD)	13.4 (4.5)	
Comorbidities	Mean (SD)	2.3 (1.8)	
Total health care contacts	Mean (SD)	17.7 (16.1)	

*Note*: The table displays hazard ratios (HRs) and 95% confidence intervals (CIs) for each variable. The null effect line (HR = 1) represents no effect. Values greater than 1 indicate an increased risk of PICU admission, while values less than 1 indicate a decreased risk compared with the reference category.

The adjusted model for PICU admission based on CCC classification showed that children with acquired condition HR = 0.46 (0.37–0.58; *p* < .001) and neurological‐congenital‐genetic HR = 0.35 (0.29–0.43; *p* < .001) had significantly lower odds of PICU admission than those with malignancy (Table 3). On the other hand, younger age of the child HR = 0.93 (0.92–0.95; *p* < .001) and higher maternal educational level: secondary or high school HR = 0.66 (0.55–0.79; *p* < .001) act as protective factors for PICU admission (Table 3).

Conversely, the need for surgical intervention HR = 1.58 (1.34–1.86; *p* < .001) and the use of any medical device HR = 1.81 (1.54–2.13; *p* < .001) almost doubles the likelihood of PICU admission, while having a transplant HR = 3.74 (1.91–7.34; *p* < .001) quadruples that risk. The presence of comorbidities HR = 1.05 (1.01–1.10; *p* = .016) and total health care frequentation HR = 1.02 (1.02–1.03; *p* < .001) also have a significant effect, albeit more discrete than the other factors.

### 5.5 Length of stay in the PICU

Regarding the CCC classification, the LOS in the children with malignancy was (median 1.00;(0–7) 0 days for patients with acquired condition (0–1) and 0 days for patients with neurological‐congenital‐genetic condition (0–2). Statistically significant differences were only found between the malignancy group and the other two groups (z = 10.70; *p* = .005). Furthermore, no significant differences were observed concerning the comorbidity groups (*z* = 0.36; *p* = .837). Additionally, there was no significant correlation between the number of comorbidities and the length of PICU stay (*r* = 0.092; *p* = .147). Similarly, the general linear regression model did not reveal any significant differences in LOS in the PICU (*F*(2, 244) = 1.047; *p* = .352).

**FIGURE 2:**
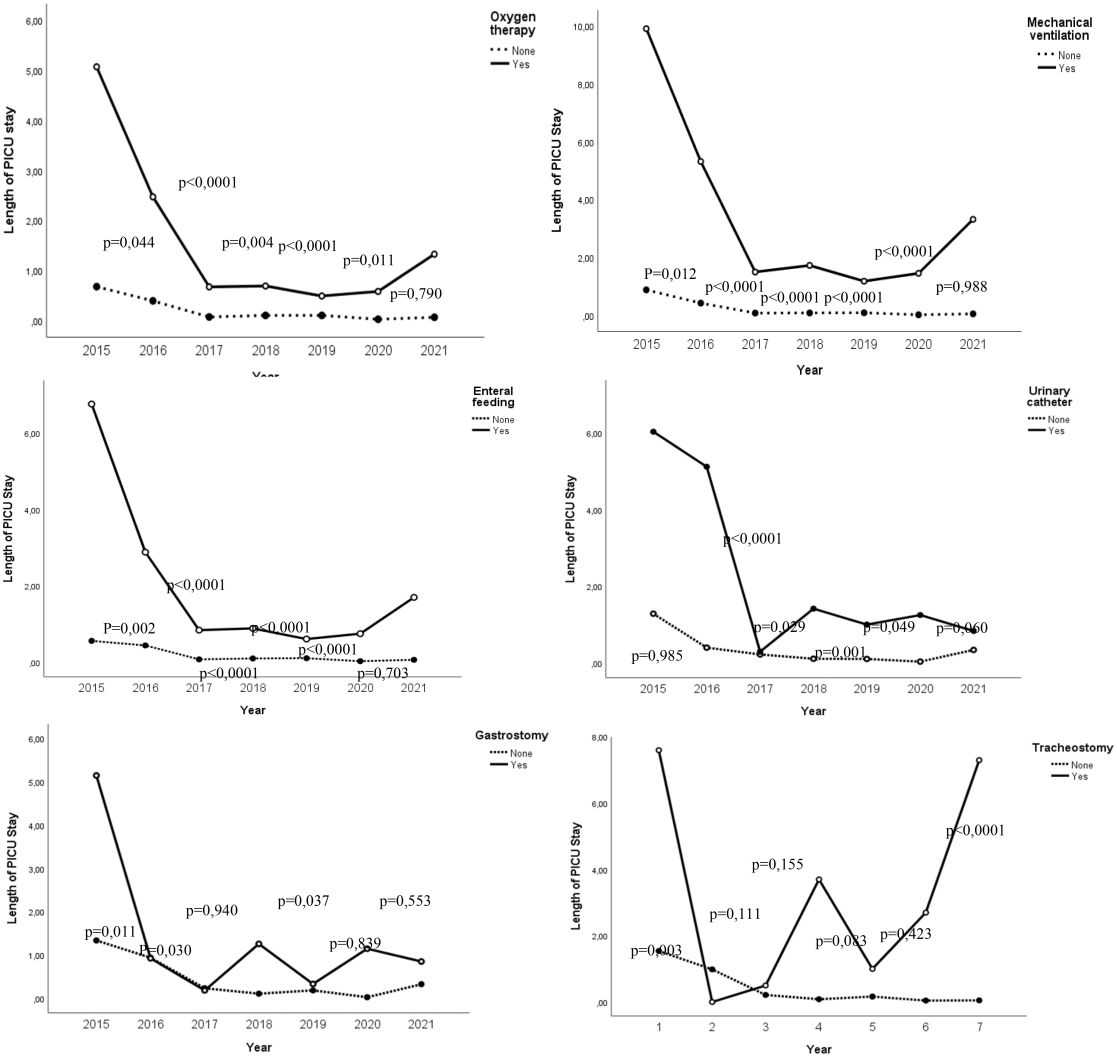
Length of stay in paediatric intensive care unit by medical devices, 2015–2021.

Significant differences were observed in children with medical devices at home, with a mean difference of −6.1 days (95% CI: −9.5 to −2.9; *z* = −3.7; *p* < .001). A detailed description of these differences by the type of device is available in Figure 2.

## 6 DISCUSSION

The aim of this study was to identify risk factors that can differentiate CMCs who are likely to require admission to the PICU.

Overall, the results show a predictive pattern of PICU admission in CMCs associated with the CCC classification, comorbidities, home medical devices, surgical procedures (including transplants) and higher health care service use as precipitating factors. Conversely, socio‐demographic factors such as the age of CMC and the mother's educational level act as independent protective factors against PICU admission.

Regarding mortality, our results show a low incidence (1.1%) as well as an extended median survival time since the diagnosis of the CMC condition (7.1 years). Previous longitudinal studies have shown similar or higher mortality rates (2.8%–5.4%), although the distribution of comorbidities in the study that obtained higher figures was different to that observed in our study.^13^

The pattern of PICU admissions for CMCs has changed, with an increasing frequency of complex chronic disease patients utilizing these services.^14^ Children who usually have medical devices represent an advanced stage of complications. Moreover, PICU admission can lead to the introduction of additional medical devices.^15^, ^16^ Our results show how the use of medical devices increases the likelihood of being admitted to the PICU (HR 1.81).

In the Cox regression model, we identified that children who underwent surgical interventions had a higher risk of PICU admission and were more likely to have undergone previous surgical interventions or transplantation. According to the literature, transplantation is recognized as a factor influencing not only PICU admission but also survival,^17^ possibly due to associated complications. In our study, the dominant disease group was neurological‐congenital‐genetic condition, although malignant diseases had the highest representation in PICU admissions. It is important to clarify that while malignancy and the presence of multiple comorbidities significantly impact the LOS in the PICU, malignancy itself also increases the likelihood of PICU admission compared with other conditions. Children with malignancy tend to have longer median lengths of stay in the PICU, reflecting the increased complexity and resource needs associated with these conditions.^18^

The inverse association detected between the educational level of mothers and PICU admission is remarkable, acting as a protective factor. In this regard, it is possible that mothers with higher levels of education are more likely to engage in health‐beneficial behaviours for their children, including health service utilization.^19^ Additionally, the father's educational level may also have a positive effect,^20^ or show no significant relationship with health outcomes,^21^ consistent with our findings. A higher level of parental education could also influence the health literacy of the children, enhancing health‐related skills and management, thereby potentially reducing the need for PICU admissions. Similarly, higher educational attainment may be associated with improved access to supportive resources.^22^ Based on these results, from a care perspective, individualized assessments would be necessary to guide the deployment of educational interventions, adapted to socio‐economic factors and follow‐up with sufficient anticipation and evaluate their impact on PICU admissions. Paediatric nurses, advanced practice nurses and the rest of the health care team should systematically incorporate this approach when providing care to CMCs and their families to individualize specific interventions (support, guidance, education) aimed to prevent admission to the PICU.^23^

### 6.1 Limitations and recommendations for further research

This study has several limitations common to follow‐up studies in PICUs. One significant limitation is the potential confounding effect of not distinguishing long‐term outcomes resulting from the underlying disease, complication of acute illness or a complication generated in intensive care. Additionally, the relatively small sample size for certain CCC categories may have limited our statistical power to detect significant differences, despite efforts to mitigate this limitation through grouping.

Another limitation is the potential influence of unmeasured confounding variables, such as variations in clinical practices among different paediatric ICUs and resource availability, which could impact ICU admission and outcomes. To address these limitations, future research should focus on larger, multicentre studies to confirm findings and explore identified risk factors more comprehensively. Prospective studies with standardized data collection methods and consideration of additional potential confounding variables would help clarify the relationships observed in this study.

## 7 IMPLICATIONS AND RECOMMENDATIONS FOR PRACTICE

Our study findings have important implications for critical care nursing practice. Nurses should prioritize proactive management of comorbidities in children with CCCs to reduce the likelihood of PICU admission. Providing education and support to families, particularly those with lower education levels, can empower them to engage in effective health care utilization. Standardizing protocols and promoting interdisciplinary collaboration within PICU settings can enhance the consistency and quality of care delivery. By addressing these key areas, nurses can optimize care for CMCs and minimize the need for PICU admission.

## 8 CONCLUSION

Our study presents the derivation of some key factors behind PICU admissions in CMC, shedding light on the interaction among the presence of malignancy, comorbidities, home medical devices, surgical procedures (including transplants) and higher health care service use as precipitating factors. On the other hand, the protective role of parental educational level suggests a complex interplay between socio‐economic determinants and PICU utilization outcomes. Furthermore, this finding points at potential personalized care strategies, such as tailored educational interventions. Nurses who deliver care to CMCs could implement this individualized approach to assist both the children and their parents, aiming to reduce the frequency of admissions to PICU through innovative supportive interventions.

This study addresses the derivation stage for the development of a clinical prediction rule. Additional studies will have to be deployed to obtain evidence of reproducible accuracy in multiple clinical settings to calibrate its overall performance, calibration and discriminative power.

## FUNDING INFORMATION

This research received no specific grant from any funding agency in the public, commercial or not‐for‐profit sectors. Funding for open access charge: Universidad de Málaga/CBUA.

## ETHICS STATEMENT

This study used data from clinical records and did not use direct data collection from patients or their families; therefore, the ethics committee of Granada considered it exempted from the need for approval and informed consent, confirming the validity of the previous study's approval (No. 0655‐N‐16).

## Data Availability

The data that support the findings of this study are available from the corresponding author upon reasonable request.
